# From problem people to addictive products: a qualitative study on rethinking gambling policy from the perspective of lived experience

**DOI:** 10.1186/s12954-018-0220-3

**Published:** 2018-04-06

**Authors:** Helen E. Miller, Samantha L. Thomas, Priscilla Robinson

**Affiliations:** 1 Centre for Population Health Research, School of Health and Social Development, Faculty of Health, Deakin University, Locked Bag 20000, Geelong, VIC 3220 Australia; 2 School of Public Health and Human Biosciences, Faculty of Health Sciences, La Trobe University, Melbourne, Australia

**Keywords:** Gambling, Harm, Stigma, Policy, Discourses, Personal responsibility

## Abstract

**Background:**

Previous research has shown that government and industry discussions of gambling may focus on personal responsibility for gambling harm. In Australia, these discussions have largely excluded people with lived experience of problem gambling, including those involved in peer support and advocacy.

**Methods:**

We conducted 26 in-depth interviews with people with current or previous problem gambling on electronic gaming machines (EGMs) involved in peer support and advocacy activities, using an approach informed by Interpretive Policy Analysis and Constructivist Grounded Theory.

**Results:**

Participants perceived that government and industry discussed gambling as safe and entertaining with a focus on personal responsibility for problem gambling. This focus on personal responsibility was perceived to increase stigma associated with problem gambling. In contrast, they described gambling as risky, addictive and harmful, with problem gambling resulting from the design of EGMs. As a result of their different perspectives, participants proposed different interventions to reduce gambling harm, including reducing accessibility and making products safer.

**Conclusions:**

Challenging the discourses used by governments and industry to describe gambling, using the lived experience of people with experience of gambling harm, may result in reduced stigma associated with problem gambling, and more effective public policy approaches to reducing harm.

## Background

Every year, approximately 0.5 to 1% of Australian adults experience problem gambling [1], with similar rates observed across jurisdictions with legalised gambling, particularly legalised electronic gaming machines (EGMs) [2]. Traditional understandings of problem gambling have recently been challenged by public health approaches which focus on a broad spectrum of harm associated with gambling [3–6]. These different approaches imply different ways of understanding gambling, which in turn imply different policy responses to reduce and prevent harm for consideration by researchers, public health advocates and governments. These contrasting ways of understanding gambling are also influenced by government and EGM industry discourses which focus on personal responsibility for gambling harm [7–12], with some research suggesting that these frameworks may entrench gambling harm, particularly through stigma [13].

Policy decisions by government, and public debate over policy, are known to be informed by the discourses which underpin public discussions. Yanow describes the process whereby discursive practices create a shared sense of meaning for groups involved in policy, creating “interpretive communities” which share common language and practices ([14], p. 10). The discourses associated with these interpretive communities “entail courses of action” ([14] p. 12): the discursive construction of an issue is directly linked to the policy approaches recommended by different groups. Several commentators have documented government and industry discourses about gambling [7–9, 11, 15]. These authors have examined how a focus on personal responsibility in government and industry discourses may be used to avoid meaningful reform in gambling by placing the emphasis on the behaviour of people experiencing problem gambling, rather than government policy or industry behaviour. However, the alternative discursive construction of gambling by people with lived experience of problem gambling, particularly those who are involved in policy debates through peer support and advocacy, have not been examined.

This is particularly important because a focus on personal responsibility may cause harm for people with experience of problem gambling. Jones et al. argued that “a marked [stigmatised] individual is treated better when he or she is not judged to be responsible for the condition” ([16] p. 57). Several studies have demonstrated that personal responsibility approaches in public policy approaches and campaigns may contribute to the stigma experienced by people who are obese [17, 18], have a mental health problem [19] or have experienced gambling harm [12, 13]. For example, Miller and Thomas [13] found that participants found the discourse of responsible gambling promoted by governments and industry contributed to negative stereotypes of people experiencing problem gambling as “irresponsible”, created norms of gambling behaviour which participants were unable to fulfil, leading to feelings of moral culpability, causing feelings of self-blame and self-stigma. Similarly, in another study of 100 gamblers, Miller and Thomas [12] found that discussions of responsible gambling led participants to hold negative stereotypes of people experiencing problem gambling as irresponsible or “undisciplined” and to focus on personal responsibility for problem gambling. Despite this, personal responsibility framing is still the most dominant approach in gambling-related campaigns and public health messaging strategies [20].

How then to reshape public discussions about gambling in ways which encourage effective policies to reduce harm? As with other areas of health [21, 22], one strategy may include the perspectives of people impacted by gambling harm. In Australia, while some consumer forums and advocacy initiatives have started to provide opportunities for people to speak about their experiences with gambling, people who have been impacted by gambling harm have been largely marginalised (or completely excluded) from the policy-making process used by governments. This is concerning, given that people impacted by gambling harm may have important knowledge based on their lived experience of how gambling discourses impact on gamblers and may have perspectives on how more effective policies can be formed by their experiences of gambling [14]. People with experience of problem gambling in particular form an important interpretive community, whose understanding of gambling may have important implications for public policy. However, while in other areas of health, consumers’ right to influence the language used to describe conditions [22], and their inclusion in the development of health service and government policy responses [23, 24], has been considered important, limited attention has been given to consumer perspectives in gambling.

This study aims to address this gap by examining the views of people with lived experience of problem gambling with EGMs on public discussions of EGM gambling, and what implications this has for appropriate interventions to reduce or minimise gambling harm. We focused on people working in advocacy and peer support, as we thought they had a unique perspective on problem gambling discourses, and significant local knowledge of gambling policies and their impacts. We explored four research questions:

- RQ1: How do participants working in peer support and advocacy perceive government and industry discussions of gambling?
- RQ2: What implications do government and industry discussions of gambling have for participants?
- RQ3: How do participants discuss gambling?
- RQ4: What approaches to reducing EGM harm do participants recommend?

## Method

This study used an approach based on Interpretive Policy Analysis [14]. Users of Interpretive Policy Analysis recognise that government policy is developed through a contestation of meaning between different actors and examines “through what processes policy meanings are communicated and who their intended audiences are, as well as what context-specific meanings these and other ‘readers’ make of policy artefacts” [14]. An Interpretive Policy Analysis is fundamentally concerned with the values held by different groups in a debate, the language used in policy discussions and the ways of understanding that may be specific to those most affected by a policy discussion. In this article, we examine how people with an experience of problem gambling interpret discourses about problem gambling and how their interpretations are in conflict with government and industry perspectives.

Yanow highlights the importance of “the very mundane, expert understanding of and practical reasoning about local conditions derived from lived experience” ([14] p. 5). The attitudes and opinions of people who have experienced gambling harm has not generally been taken into account in policy development in Australia. In this article, we focus on the lived experience of problem gambling on EGMs for people who were involved in peer support and advocacy. We used an interpretive approach informed by Constructivist Grounded Theory [25, 26] to conduct in-depth interviews with people with experience of problem gambling. We received ethical approval from the university Human Research Ethics Committee prior to the commencement of the study.

### Recruitment

Our original study was designed to examine experiences of stigma in a wide range of people who had personal experiences of problem gambling, with a focus on how stigma occurs in the context of government and industry policies on gambling. We interviewed participants who were living in Australia and were fluent in English. We conducted several initial interviews with people involved in peer support and advocacy activities, to pilot our interview guide. In these interviews, we found that people working in peer support and advocacy had unique perspectives relevant to gambling policy development and we decided to focus only on people with an experience of problem gambling who were involved in peer support and advocacy for this study. As there are some differences in the regulatory and industry structures, as well as levels of stigma, which exist for different gambling products, we elected to focus only on people who had had a problem with EGMs.

Recruiting for the study was difficult, as even people involved in peer support and advocacy can be reluctant to talk about their gambling experiences as a result of stigma. In addition, there are only a small number of people with lived experience working in peer support and advocacy in Australia. We used both snowball and convenience sampling to recruit participants. To reach people working in peer support and advocacy organisations, we asked that information be distributed through the relevant organisations. We used online and email strategies to contact people working independently. We also asked participants to pass on the details of the research team to others who might be interested in participating.

### Data collection

We conducted 26 semi-structured, audio-taped interviews from May 2015 to July 2016. Each interview lasted between 45 and 70 min and could be conducted in person (*n* = 12) or by phone (*n* = 14). Participants were provided with a participant information sheet and provided verbal consent to participate in the study and for the interview to be audio-recorded. Participants were first asked a range of demographic and socio-economic questions. We also assessed their current experience of gambling problems using the Problem Gambling Severity Index (PGSI) [27]. A series of open-ended questions was asked focusing on the themes of experiences with gambling, norms for gambling behaviour, public discussions of gambling, responsible gambling, community views of people with gambling problems, responding to gambling problems and experiences of peer support and advocacy. In exploring public discussions of gambling, we asked about what messages governments, industry and media promote about gambling and what participants would like to change about these messages. We also asked participants what the 'top three' interventions to prevent problem gambling would be.

### Data interpretation

We used a thematic approach to data interpretation informed by Interpretive Policy Analysis [14, 28]. We were concerned with how participants resisted and contested government and industry understandings of problem gambling, and the way that their alternate understandings informed their proposed responses. We used the constant comparative method, utilising NVivo 16 for data management, to identify different themes both within participants’ transcripts and compare and contrast themes between different participant’s data [29]. The lead author led the data interpretation by reading transcripts line by line and assigning initial codes, with the co-authors also reviewing the data and assigning codes. The first two authors met regularly to discuss and refine the initial interpretations. We conducted data interpretation alongside data collection, and emerging themes from the data analysis were explored in more detail in subsequent interviews. Our recruitment process was driven by data interpretation, as we sought out participants who would be able to provide rich data on emerging themes. We ceased recruiting participants when we found we had enough data to explore a number of themes relating to the key research questions.

Consistent with participatory approaches [30], we wanted to ensure that our findings and interpretations of data were consistent with the views of people with an experience of problem gambling. We therefore asked four leaders from consumer and carer organisations, all with lived experience of gambling harm, to check and comment on our initial interpretations. This group endorsed the interpretations we outline in this paper.

## Results

### Sample characteristics

The characteristics of the sample are shown in Table 1. Fifteen participants were female and 11 were male, most had received at least a year 12 education (meaning they had completed secondary school), and half (*n* = 13) had a tertiary or university qualification. Three participants were involved in peer support programs, 14 were involved in advocacy, and nine were involved in both activities. When asked the Problem Gambling Severity Index [27], eight participants scored as having no current problems with gambling, one was at low risk, 11 were at moderate risk, and six were categorised as problem gamblers.

**Table 1:** Sample characteristics

Characteristic	Participants (*n* = 26)	Percentage
Gender		
Female	15	57.7
Male	11	42.3
Age		
25–34	4	15.4
35–44	3	11.5
45–54	4	15.4
55–64	10	38.5
65+	5	19.2
Level of education		
Less than year 12 (did not complete secondary school)	5	19.2
Year 12 (completed secondary school)	2	7.7
Trade or TAFE qualification (vocational qualification)	6	23.1
Tertiary (university) qualification	13	50.0
Employment		
Employed full time	8	30.8
Employed part time	6	23.1
Unemployed and looking for work	5	19.2
Not employed and not looking for work	7	26.9
Country of birth		
Australia	18	69.2
Elsewhere	8	30.8
Problem Gambling Severity Index		
Non-problem gambling or non-gambler	8	30.8
Low-risk gambling	1	3.8
Moderate-risk gambling	11	42.3
Problem gambling	6	23.1
Individual advocacy and/or peer support		
Advocacy	14	53.8
Peer support	3	11.5
Both	9	34.6

Four key themes emerged from the data. A summary of the results is shown in Table 2.

**Table 2:** Participants’ perspectives on government and industry framing of gambling and suggestions for alternative framing

Government and industry perspectives	Participants’ perspectives
Gaming machines as: • Safe • Entertaining • Beneficial to the community	Gaming machines as: • Risky • Designed to addict • Harmful for the community
Problems from gambling as: • Rare • Based in gambler behaviour • Caused by 'problem gambling' and 'problem gamblers'	Problems from gambling as: • Common in people who use EGMs regularly • Due to the addictive nature of machines • Caused by 'gambling addiction' • Happening to 'people with gambling problems'
Solutions for problem gambling: • Based on the personal responsibility of gamblers	Solutions for problem gambling: • Based on public health • Require changes to government and industry behaviours
Policy responses: • Focus on treatment • Focus on individual behaviour	Policy responses: • Reducing accessibility to EGMs • Making products and venue environments safer • Educating the community about harm
Implications for gamblers: • Stigma • Blame	Implications for gamblers: • Reduced stigma and blame • Reduced harm

### Theme one: safe and entertaining—the minimisation of EGM harm

Participants believed that governments promoted a perception of EGM gambling that ignored or minimised the harm associated with this product. For example, one participant said that the government’s position was that “there’s no problem” with gambling associated with EGMs. However, governmental perspectives were seen by a few participants as influenced by the way the community understood the issue of gambling. One participant suggested that government attitudes to EGM gambling was in fact driven by a lack of understanding of the harm associated with problem gambling, because the community “don’t have any really clear indication about the costs”. As a result of this lack of understanding of harm, this participant argued that the community failed to prioritise gambling as an issue, because:

[If people are asked] what are the most important issues in your community, you know, gambling doesn’t rate, or problem gambling…so if the government looks at this, and see…why would I invest and try to do something for that, if nobody cares about it. (Female, PGSI 0).

Participants also thought that the EGM industry discussed EGM gambling in a way that minimised harm and emphasised fun and entertainment. Some participants argued that the EGM industry did not discuss problem gambling at all, preferring to focus on the positive elements of EGM gambling emphasising that “gambling is fun, that they are an entertainment industry”. One participant reflected on industry discussions of problem gambling in the media as focusing on “a very, very small number of people that actually have this problem”, or even “that there isn’t such a thing as gambling addiction”. By minimising or even ignoring the scale of harm from problem gambling, industry was seen as presenting EGM gambling as “a safe thing to do”. Participants also thought that industry framed EGM gambling as a positive for the community, and emphasised the benefits of the gambling industry, such as donations to community groups, as outweighing the harms. One participant commented that the EGM industry argued that “all the good that they do in the community absolves them of anything bad that comes from the poker machine industry”.

### Theme two: a focus on problem people rather than problem products

Participiants perceived that one way governments minimised attention given to harm from problem gambling on EGMs was to emphasise that gambling harm came from “a few individuals” with severe problems. Participants felt that governments emphasised individual responsibility for problem gambling and, in discussions of problem gambling, tended to “blame the victim”. One participant described the government’s position as “it’s your problem, you wanted to gamble”. Participants also perceived that the government also had a strong focus on encouraging help-seeking and treatment, which was also perceived in individual terms. For example, while there were extensive campaigns about problem gambling, it was “up to the individual” to seek help. This meant that government solutions for problem gambling were largely influenced by a belief in the personal responsibility of gamblers.

At the same time, the gambling industry focused on individuals with “addictive personalities” rather than “addictive” products. Participants argued that industry’s position was that “the people for whom gambling is a problem are the problem, not the industry, not the machines”. They contrasted the focus on individual behaviour with the inherently addictive nature of EGMs. One participant said:

People don’t realise that the machines have terrible odds. That they’re designed the way they are to be addictive and to draw you in. So the industry tends to be like ‘well, we can’t focus on that because that will deter people from playing’. So it just takes away from that and just puts it onto the user. So our product’s not addictive. You’ve got a problem if you get addicted. (Male, PGSI 0).

Participants argued that the industry preferred to emphasise the responsibility of gamblers when discussing problem gambling, rather than the nature of their products, and put the “onus for the problems on the individuals”.

### Theme three: shifting the focus from individuals to products

In their work, advocates aimed to “reshape the narrative” around EGMs, to increase the focus on products and the behaviour of industry, rather than individual gamblers. Participants wanted to focus on an alternative perspective on problem gambling and to “treat it a bit like a public health issue”. In particular, participants were keen to stress that EGMs are an “addictive product”, which were “designed to addict you”. They argued that the community needed to be made aware that EGMs could be addictive:

I think people need to understand that they are designed to captivate you, and if something’s going on in your life, that that captivation makes it easy for you to go back to, to escape whatever’s happening. And there’s no education about it, no one understands how addictive it is. (Female, PGSI 10).

Almost all the participants discussed addiction at some point in the interview. Participants argued that focusing on the potential for addiction would reduce stigma specifically associated with problem gambling, by reducing blame of people who developed problems with EGMs. One participant said:

I think it needs to be known as an addiction, just for it to get, for people to respect it a bit more, and not look down on it so much…people just think you’re an idiot, and it’s your own fault for getting stuck in that, and losing all that money. I think while it’s not an addiction… the general public will look at it, look at people who have it, as… stupid, that sort of thing. Or, that, ‘you’re an idiot.’ (Male, PGSI 0).

Participants generally thought the community was not adequately educated about the way EGMs were designed, or the potential for addiction to the machines, which led to assumptions that gamblers were “stupid”, were “lazy” or had “no values”. Participants described the need to put an emphasis in public discourse on addictive products, rather than individual gamblers. One participant emphasised that the community needed to understand that machines were addictive, in order to reduce the focus on personal responsibility of the individual gambler:

It’s not just the person’s fault that they have a problem with gambling on the pokies, that the machines are deliberately designed to trap and trick us…it’s quite normal for a person to get hooked. (Female, PGSI 0).

Participants also wanted the community to have a clearer understanding that gambling was associated with significant risk, and perceived that this would counter government an industry discussions of gambling, which minimised the amount of harm associated with gambling. Participants described that there was insufficient information about the harms that could be caused by gambling, and wanted to highlight the harms that arose from gambling to others in the community. One participant argued that society emphasised the harmful consequences of alcohol and drugs, but that “we don’t really do the same” for gambling. Participants felt that the current ways of framing gambling, which focused on fun and entertainment, led gamblers to have a reduced perception of risk associated with gambling. One participant said:

It is marketed in such a way that it’s just entertainment, you know, so why would people think that it could cause them any kind of harm? There’s no education… I mean, I grew up knowing that people could be addicted to drugs and alcohol, but nobody ever told me that gambling could ruin your life. (Female, PGSI 5).

Some participants wanted significant changes to the language used to describe problem gambling, particularly around the term “problem gambler”. Most participants did use the term problem gambler in their discussions; however, they generally did not use it to label their own behaviours. Some participants rejected the term problem gambler as stigmatising. Participants said this language “made me feel like I was the problem” and “makes the public think that they’re dealing with somebody who is a problem”. One participant linked the use of language such as labelling a person a problem gambler to broader industry attempts to focus on the behaviour of gamblers, rather than the addictive nature of EGMs. She said:

The industry really coined that phrase 'problem gamblers', like they [gamblers] are the problem, the machine isn’t, when they know full well that it was designed to addict people. (Female, PGSI 5).

### Theme four: the implications of alternative framing—recommendations for preventing harm

Because participants believed there needed to be a greater focus on the addictive nature of the product, many of their suggestions focused on reducing the access the community has to EGMs. One of the most common responses to our question on responses that would prevent harm was simply to “get rid of them [EGMs]”. Participants thought that there were too many machines, and they were too easily accessible when located in suburban areas. Many participants favoured removing machines from hotels and clubs, and retaining only machines at casinos. One participant said that machines were “out of sight, out of mind” if they were located in a casino, away from her local area, and she would “just go and do something else”. Participants also wanted to be able to go to venues in the community without being exposed to gambling and were concerned that there were not many options available for recreational activities that did not involve gambling. Some participants also suggested other reductions in accessibility, such as reductions in opening hours.

Similarly, participants who focused on EGMs as addictive products had suggestions about how these product could be made less harmful. Participants made a series of suggestions about ways to make the product safer. Most common was a mention of $1 maximum bets, which limits the amount that can be lost per spin to $1. One participant said:

If it was just $1 you’d wonder whether they’d stick at it. And they certainly wouldn’t lose so much. To be able to lose $1000 in an hour is not a safe practice. (Female, PGSI 4).

Some participants also discussed mandatory card-based systems to minimise gambling-related harm. Often, this involved a discussion of setting mandatory limits, but these participants did not always focus on limit setting. Sometimes, they thought these systems should be used as reliable mechanisms for excluding people experiencing problem gambling from venues, perhaps also requiring biometric data such as a fingerprint. A participant explained:

One of the things I think should be that all pokie rooms have card access so you can’t get into one without card access; they’re very secure places and if you’re a problem gambler, you are restricted to either times that you can go in there or you can’t go in there at all. (Female, PGSI 0).

Other recommendations for reducing harm include reducing “free spins”, “incentives” (loyalty programs), “false wins” (losses disguised as wins) and the rate at which machines spun. One participant also discussed removing ATMs from venues. Finally, one participant suggested that venue environments should be changed to remove all chairs in front of machines. She argued:

I think removing the chairs means you can’t settle in front of a machine… the idea is you’ve got to move people off…stop people getting comfortable. (Female, PGSI 0).

In response to government and industry minimisation of the risks associated with gambling, and participants’ desire to increase understanding of harm associated with gambling in the community, many participants discussed options for educating the community about gambling harm. Some participants felt that governments should implement hard-hitting social marketing campaigns, similar to those used in smoking, using stories of real people with experience of gambling harm to drive home the message that gambling was harmful. Participants also emphasised that it was particularly important to educate young people about the risks of gambling before they started to gamble. In particular, they wanted people with a lived experience of problem gambling to “spread the word” and be involved in educating young people and others in the community about the risks of gambling. Participants were focused on prevention approaches, rather than treatment, with one participants saying:

It’s worth reframing the discussion away from treatment - treatment’s important, but it shouldn’t be the be all and the end all, we need to make sure we take on the problem before it happens. (Male, PGSI 5).

## Discussion

Before discussing the results of this study, it is important to consider the limitations of this study. Our sample is relatively small and only included people with experiences of problem gambling who were also involved in peer support and advocacy activities. Those who were not involved in these activities, or who had experienced gambling harm at less severe levels,may have a different understanding of gambling discourses. These understandings should be explored in future research. We have also not identified any differences in perceptions of gambling discourses based on the participant age, gender and ethnicity, which might be revealed in a sample with a broader range of people with experience of gambling harm. In addition, our sample are all Australian, and these findings may not reflect the situation in other jurisdictions.

This study was designed to examine how people with an experience of problem gambling working in peer support and advocacy in Australia understand gambling, and what implications their understanding of gambling have for government policy responses to reduce harm. Participants articulated quite different perspectives on gambling from what they saw as the views of government and industry, which they argued understated harm associated with gambling. Previous work examining government and industry documents confirms our participants’ view that these institutions focus on gambling as entertaining, beneficial for the community and not associated with significant levels of problem gambling [11]. This represents a significant difference from participants’ view of gambling as risky, harmful and addictive, with problems as a result of gambling being relatively common. Government and industry discourses around gambling may be the result of norms and values held within these institutions and may have complex historical causes [31]. However, previous researchers have suggested that public discussions about gambling are strategically focused on gamblers as 'flawed consumers' to reduce pressure for reform and justify the revenue government and industry make from EGMs  [9]. Participants’ views were more consistent with the views expressed by researchers focusing on the public health aspects of gambling [3, 6, 32]. They had a strong focus on harm and, consistent with this emerging literature, thought that harm was common and affected a large number of gamblers.

Participants were also critical of the focus on personal responsibility in government and industry discourses related to problem gambling. Personal responsibility discourses, including both discussions of 'responsible gambling' and also a focus on promoting individualised help seeking, are common in social marketing campaigns and other government responses to problem gambling [11, 20]. Participants argued that a personal responsibility approach contributed to the stigmatisation and blame of people with gambling problems. This is consistent with previous research, which has shown that a focus on personal responsibility, particularly through discourses about responsible gambling, contributes to the felt and enacted stigma of people with experience of problem gambling [12, 13]. In contrast to a focus on personal responsibility, participants recommended a focus on the addictive properties of EGMs, both as a way to reduce the stigma associated with problem gambling and as an approach to reducing harm. Highlighting the risk of addiction associated with a product may promote more 'responsible' behaviour due to an increased awareness of the risks associated with these products.

The way that participants in this study understood gambling also helps to explain why current government approaches to reducing harm may be ineffective. Consistent with Yanow’s discussion on the importance of shared communities of meaning in forming policy responses [14], we found that participants’ understandings of problem gambling implied particular policy responses which differed from those favoured by government and industry. Previous research has shown a strong emphasis on treatment in government approaches to problem gambling, which is informed by a focus on personal responsibility [11]. However, participants’ perspectives, which highlighted the role of EGMs and the EGM industry in creating harm, suggest that approaches to harm reduction need to be grounded in changes to the nature or availability of EGMs, or in educating the community about addictive properties associated with EGMs. Participants considered that treatment, while valuable, was not the most important way to reduce harm from gambling.

Understanding the perspectives of people with an experience of problem gambling, particularly those involved in peer support and advocacy, may be valuable in identifying effective government policy interventions. Many of the government policy responses suggested by participants in this study were consistent with approaches to harm reduction advocated as effective by public health researchers, such as maximum bets and mandatory pre-commitment [33]. However, some were new, such as the suggestion to prohibit chairs in EGM rooms. While further research is required to demonstrate whether this would be an effective way to reduce harm, the suggestion demonstrates that people with an experience of problem gambling may be able to suggest novel approaches to reducing harm from gambling, and should be encouraged to form part of the policy development process undertaken by public health advocates. There is also a key role to be played by advocacy groups in representing the views and experiences of individuals, communities, and populations who have been impacted by harmful gambling. This includes researchers and others involved in policy-making processes. This may enable individuals, their families, and friends to feel that they are supported in speaking out and also allow anonymous or pseudonymous participation in advocacy.

This study also suggests avenues to learn from other major public health issues. Some suggestions made by participants, such as hard-hitting social marketing campaigns, had strong parallels with approaches to tobacco harm minimisation [34]. It is therefore important to learn from the work already conducted in other areas of public health not only in identifying effective strategies to reduce harm but also in the need to challenge industry narratives and lobby governments to implement more effective harm minimisation strategies.

Since the early 1990s, researchers, policy-makers and other writers have been encouraged to use 'people first' language when describing people with disabilities or mental illnesses [35]. Thus, problem gambler becomes 'person with experience of problem gambling' or similar. The purpose of people-first language is to emphasise the personhood and agency of the individual involved and to avoid implying that their gambling problem is the only important aspect of their identity [36]. It has been demonstrated that using people-first language is associated with a reduction in stigmatising attitudes [37]. However, the term 'problem gambler' is still in common use in Australia in government, industry and media discourses [10, 11]. This was a concern for our participants, who felt it was linked to broader discourses which emphasise the personal responsibility of gamblers. Further research is needed with a broader range of gamblers to determine the most appropriate way to refer to people who experience problem gambling, as well as the broader group of gamblers, family and friends who may experience harm. Consideration in many instances should also be given to whether it is appropriate to discuss problem gambling or the broader concept of harm. However, at a minimum, researchers and those involved in public discussions of gambling should use people-first language.

## Conclusion

This study has highlighted the importance of integrating the views of people with an experience of problem gambling into public health approaches to reducing harm from gambling. Ignoring the perspectives of people with an experience of problem gambling has led to approaches which promote stigma, such as public discussions which focus on personal responsibility, and to a lack of effective approaches to harm reduction. Researchers and public health advocates should ensure that people with lived experience of gambling harm (including those experiencing less severe harm and also the friends and family of gamblers) are included in the advocacy and policy-making process.
